# Determinants of Vitamin A Consumption Among Children Aged 6–23 Months in Somalia: A Multilevel Analysis of SDHS 2020

**DOI:** 10.1002/fsn3.71301

**Published:** 2025-12-08

**Authors:** Abdirahman Omer Ali, Abdisalam Mahdi Hassan, Hawa Dahir Mahamoud, Nimco Mahamoud Barakale, Muhyadin Yusuf Dahir, Md. Moyazzem Hossain

**Affiliations:** ^1^ Amoud University College of Health Science School of Medicine Borama Somalia; ^2^ School of Postgraduate Studies and Research, Amoud University Borama Somalia; ^3^ Office of Community Services Amoud University Borama Somalia; ^4^ Department of Statistics and Data Science Jahangirnagar University Savar Bangladesh

**Keywords:** child health, micro‐nutrient deficiency, multilevel analysis, vitamin A deficiency (VAD)

## Abstract

Vitamin A deficiency (VAD) affects children under 5 years of age and leads to deaths predominantly in low‐ and middle‐income countries (LMICs). VAD remains a significant public health concern in Somalia. Therefore, it is necessary to explore the determinants of VAD among children under five in Somalia using country representative data. Therefore, this study investigates the determinants of adequate vitamin A (VA) consumption among Somali children aged 6–23 months, using data from the first‐ever Somalia Demographic Health Survey (SDHS) 2020. Secondary data were extracted from SDHS‐2020, and after removing the missing observations, a sample of 7344 children aged 6–23 months was considered in this study. The Chi‐squared test is used to identify the association between the target variable and covariates. A multilevel analysis was conducted on a sample of 7344 children, examining individual‐level (maternal education, wealth, breastfeeding status, VAS history) and community‐level (region, urban/rural) factors associated with good VA consumption, defined as consuming at least one VA‐rich food item in the previous 24 h. Mixed‐effects logistic regression models were used to account for data clustering. A significant proportion of children (*n* = 4488, 61.1%) did not consume VA‐rich foods. Key predictors of consuming VA‐rich foods included maternal provision of vitamin A supplements in the past 6 months (AOR = 3.1, 95% CI: 2.6–3.7) and current breastfeeding status. Children residing in the Sool region demonstrated higher odds of receiving VA, while higher maternal education and middle‐income status were associated with lower odds of receiving VA, indicating that these factors inversely impacted the consumption of vitamin A. Community‐level variance highlighted the importance of contextual factors. The findings highlight the importance of sustained VAS programs and breastfeeding promotion in combating VAD in Somalia. Mothers' education and media exposure should be increased to lessen the burden of VAD. Addressing socioeconomic disparities and tailoring interventions to regional contexts are crucial for improving child nutrition outcomes.

## Graphical abstract

About 61.1% of children did not consume VA‐rich foods in Somalia. Findings depict that Banadir (73.2%) has the highest prevalence of consuming VA‐rich foods, followed by Woqooyi Galbeed (55.0%) and Awdal (51.5%). The lowest prevalence of consuming VA‐rich foods is observed in Sool (15.8%), Sanaag (25.2%), and Lower Juba (25.2%). Moreover, children residing in the Sool region demonstrated higher odds, while higher maternal education and middle‐income status were associated with lower odds of receiving VA.

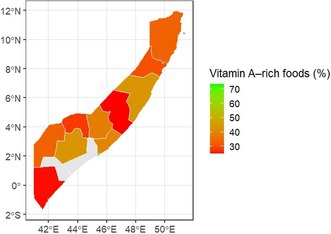

## 1 Introduction

Vitamin A (VA) is recognized as a vital fat‐soluble micro‐nutrient that plays critical roles in various physiological processes, including vision, immune defense, growth, and reproductive health (Song et al. 2023; Shastak and Pelletier 2023). On an international scale, Vitamin A deficiency (VAD) affects an estimated 190 million children under 5 years of age, leading to 1–2 million deaths annually, predominantly in low‐ and middle‐income countries (LMICs) (Song et al. 2023; Oumer et al. 2020). The Sub‐Saharan African region is particularly vulnerable, with approximately 42% of children under 5 years old at risk of VAD, a condition that accounts for nearly 6% of mortality within this age group (Oumer et al. 2020). Research conducted in various African countries, including Ghana, Malawi, and Uganda, reveals insufficient intake of Vitamin A‐rich foods, thereby highlighting the urgent need for improved nutritional interventions (Demsash et al. 2022). Contributing factors to VAD in this region include inadequate dietary quality, the prevalence of infectious diseases, and detrimental socioeconomic conditions (Wolde and Tessema 2023). However, to guarantee adequate dietary quality, it is necessary to ensure affordable healthy food (Hossain et al. 2023). In Somalia, the incidence of VAD is alarmingly high. A study undertaken in 2010 indicated that 33.3% of children aged 6–50 months were impacted, exceeding the threshold defined by the World Health Organization (WHO) for severe VAD (FSNAU n.d.). WHO has been implementing Vitamin A Supplementation (VAS) programs in Somalia since the 1990s; however, their limited coverage puts many children at risk for vitamin A insufficiency (Ali et al. 2022). Despite the WHO's endorsement of Vitamin A Supplementation (VAS) as a cost‐effective public health strategy (Solutions For Youth Employment n.d.; WHO 2025; Raut et al. 2019), the uptake of VAS in Somalia remains critically inadequate; in 2021, only 0.2 million out of the targeted 0.7 million children aged 6 to 59 months received VAS (Ali et al. 2022). Prolonged conflict, recurrent drought, forced migration, and a fragile healthcare infrastructure significantly hinder the effective delivery of essential health services, thereby exacerbating the prevailing challenges (Public Health Agency (Sweden) 2020).

Several factors influence Vitamin A consumption, including socioeconomic status (encompassing poverty and income disparity) (Dharod et al. 2011; Fekadu et al. 2015; Kalid et al. 2019), maternal characteristics (such as educational attainment and employment status) (Oumer et al. 2020), access to healthcare services (including antenatal care and place of delivery) (Oumer et al. 2020), regional disparity (Hossain et al. 2021), and community‐related factors (which include poverty levels and media exposure) (Oumer et al. 2020) Additionally, political instability and environmental challenges (such as drought) further impede the implementation of VAS programs (Ali et al. 2022). There may be an impact on vitamin A intake in certain locations, particularly for individuals residing in remote areas or those with limited access to medical facilities (Ezezika et al. 2025). Poor infrastructure and health systems in communities make it difficult to reach the most vulnerable populations, particularly children, due to factors such as distance from medical facilities, as seen in South Asia and Africa (Ezezika et al. 2025; McLean et al. 2018; Directorate of National Statistics FG of S 2020; Amimo et al. 2022) The existing literature predominantly focuses on individual factors that affect VA consumption (Oumer et al. 2020; Demsash et al. 2022; Wolde and Tessema 2023; Ali et al. 2022), and there is a lack of use of multilevel modeling to consider country representative data. Moreover, the shortcomings of previous single‐level studies are that they cannot simultaneously account for the nested structure of individual and community‐level factors. In the context of Somalia, there is a dearth of literature focusing on the VA consumption based on countrywide data. Therefore, to fill up this backdrop, this study endeavors to address this limitation by employing a multilevel analytical approach to investigate the determinants of sufficient Vitamin A (VA) intake among Somali children, utilizing data obtained from the Somalia Demographic Health Survey (SDHS) 2020. The objective of the study is to assess the prevalence and determinants of adequate Vitamin A consumption in Somali children, ultimately offering data‐driven recommendations to improve the delivery and effectiveness of VA supplementation initiatives in Somalia.

## 2 Methods and Materials

### 2.1 Data Source, Tool, and Sampling Procedure

The data for this study were sourced from the first 2020 Somalia Demographic and Health Survey (SDHS), a nationally representative survey publicly available through the MEASURE DHS program (www.measuredhs.com). With the exception of Banadir, all 18 pre‐war regions were divided into urban, rural, and nomadic categories, resulting in an initial sample size of 55. Some strata, such as the three strata in the Lower Shabelle and Middle Juba regions, as well as the rural and nomadic strata in the Bay region, were left out due to security or accessibility concerns. There were forty‐seven strata in the final sample frame. In the first step, 1433 EAs—770 urban, 488 rural, and 175 nomadic—were chosen from the 47 strata. Following the listing, 10 EAs were chosen for household surveys out of the 35 listed EAs per stratum for the urban and rural strata. After cleaning the data from the first phase, the sampling frames for the second phase were a summary of the households identified by EA. The second step involved using a probability proportional to the number of households to sample 10 EAs out of the 35 that were listed. Twenty houses in each of the ten EAs were sampled for the survey, and each household was identified by its location within the EA. To ensure the distribution of the houses interviewed for the survey within the EA sample, serialization was implemented. In total, 220 EAs and 150 EAs were assigned to the urban and rural strata, respectively. In the third stage, an average of 30 households was chosen from each EA's listed households, resulting in a total of 16,360 households from 538 EAs covered (220 EAs in the urban, 147 EAs in the rural, and 171 EAs in the nomadic) out of the 545 EAs sampled. With a probability proportionate to the expected number of families, ten EAs (in this case, TNS) were chosen from each nomadic stratum in nomadic areas. The specific sampling procedures and weights used to account for the complex survey design are detailed in the SDHS report (Directorate of National Statistics FG of S 2020). Data collection tools included standardized questionnaires administered by trained interviewers using Computer Assisted Personal Interviewing (CAPI) techniques, ensuring data quality and consistency across the survey.

### 2.2 Sample Size

Data for this study were sourced from the 2020 Somalia Demographic and Health Survey (SDHS) Kids' Recode (KR) file (Directorate of National Statistics FG of S 2020). Our study population initially comprised all children recorded in the dataset. To focus on the target age group for Infant and Young Child Feeding (IYCF) practices related to vitamin A consumption, we selected children aged 6–23 months at the time of the survey who were reported to be living with their mothers (or primary caregivers who responded to the child's questionnaire). This approach, including all eligible children in the age range rather than only the youngest child per mother (as is common for some descriptive IYCF tables in DHS reports), was chosen to maximize the sample size for our multivariable regression analysis of determinants. From this initial selection of children aged 6–23 months, we excluded cases with missing data on the primary outcome variable (consumption of VA‐rich foods) and any of the independent variables included in our final multilevel logistic regression models. A weighted sample size of 7344 children was used in the analysis.

### 2.3 Outcome Variable

Children aged 6–23 months living with their mother who consumed at least one vitamin A‐rich food item from the standard list used in Demographic and Health Surveys for Infant and Young Child Feeding (IYCF) indicators in the 24 h preceding the interview were categorized as ‘consumed VA‐rich foods’ (coded as “1”). Children who did not consume any of these food items in the 24 h preceding the interview were categorized as “did not consume VA‐rich foods” (coded as “0”). This list includes: (1) eggs, (2) pumpkin, carrots, squash (yellow or orange inside), (3) any dark green leafy vegetables, (4) mangoes, papayas, or other vitamin A‐rich fruits, and (5) liver, heart, or other organ meats. This definition aligns with the official SDHS 2020 report's methodology for this indicator and addresses the discrepancy identified with earlier versions of our definition.

### 2.4 Variable of Study

The independent variables included in this study were selected based on a comprehensive literature review, their relevance as established in studies on vitamin A consumption in LMICs, considerations of self‐efficacy, and data availability within the SDHS 2020 dataset. The existing literature has identified key socioeconomic, demographic, and behavioral factors as potential determinants. Our conceptual framework considered both individual‐level (e.g., maternal education, wealth, child's age) and community‐level (e.g., region of residence, urban/rural status) influences. Data availability within the SDHS dataset further informed the selection, prioritizing variables that aligned with our research questions and theoretical framework.

One such key socioeconomic variable was the household wealth index. As provided in the SDHS 2020, this index is originally categorized into five quintiles: poorest, poorer, middle, richer, and richest, with each quintile designed to represent approximately 20% of the households in the sample. For the purpose of this analysis, these five quintiles were reclassified into three broader socioeconomic categories: ‘Lower income’, ‘Middle’, and ‘Higher income’. This reclassification was performed to enhance the interpretability of the findings and to ensure adequate sample sizes within each category for robust statistical modeling. The reclassification was done as follows:

The **‘**Lower income**’** category was created by combining the original ‘poorest’ (bottom 20%) and ‘poorer’ (next 20%) quintiles. The **‘**Middle**’** category was created by combining the original ‘middle’ (middle 20%) and ‘richer’ (next 20%) quintiles.

The **‘**Higher income**’** category comprised the original ‘richest’ (top 20%) quintile. This reclassification approach resulted in the distribution observed in our study sample (Lower income: 43.45%, Middle: 39.68%, Higher income: 16.87%), where slight deviations from a perfect 40%/40%/20% split are attributable to the specific distribution of assets within the Somali context and the application of survey weights.

Other independent variables considered at the individual‐level included maternal age, maternal education, marital status, media exposure, preceding birth interval, child's current age, parity, history of giving vitamin A supplement in the last 6 months, place of delivery, number of ANC visits, breastfeeding status, source of drinking water, use of drugs for intestinal parasites during pregnancy, and perceived distance to health facility. Community‐level variables included region of residence and type of residence (urban/rural/nomadic).

In the bivariable multilevel mixed‐effects logistic regression analysis, variables that demonstrated statistical significance at a *p*‐value of 0.2 were selected for further analysis. These significant variables were considered for inclusion in the subsequent adjustments to the individual and community‐level models.

### 2.5 Data Management and Analysis

Data were cleaned, managed, and analyzed using STATA version 17.0. The analysis used data from the SDHS 2020 Kids' Recode (KR) file. To account for the complex multi‐stage stratified cluster sampling design of the SDHS and to ensure the results are nationally representative, survey weights were applied. The mother's individual sample weight (variable V005 from the Women's Recode, available for each child in the KR file) was used. This weight was divided by 1,000,000 to create the probability weight variable (pweight) for use in the analysis. The survey design was specified in STATA using the svyset command, defining the primary sampling unit (PSU, typically variable V021, which is the DHS cluster number corresponding to V001 from the household dataset), the stratification variable (typically V022 or a derived stratum variable, e.g., sostrat), and the calculated probability weight. A two‐level mixed‐effects logistic regression analysis (melogit command with the pweight option in STATA) was employed to account for the hierarchical structure of the data (children [level 1] nested within communities/PSUs [level 2]) and the binary outcome variable (consumed/did not consume VA‐rich foods). The probability weights were incorporated into these multilevel models to ensure weighted parameter estimates that are representative of the Somali population of children aged 6–23 months.

### 2.6 Multilevel Analysis

Given that the outcome variable was binary (‘consumed VA‐rich foods’ coded as 1, or ‘did not consume VA‐rich foods’ coded as 0), a two‐level mixed‐effects logistic regression analysis was appropriate. This approach accounts for the hierarchical nature of the SDHS data, where children (level 1) are nested within communities/PSUs (level 2). As described previously, the analyses incorporated survey weights derived from V005 (mother's sample weight) and accounted for the PSU (V021/V001) and strata (e.g., V022) survey design features. This multilevel modeling approach also inherently addresses potential non‐independence of observations, such as multiple children from the same household or community, by estimating community‐level random effects. Initially, the individual and community‐level variables were assessed independently in the bivariable multilevel logistic regression model. This analysis aimed to identify any potential associations between these variables and the consumption of VA‐rich foods.

### 2.7 Model Building and Selecting the Best‐Fitted Model

The four models (Model I–IV) containing variables of interest were fitted using STATA version 17.0 to check and select the most suitable model. We consider, (1) Model 0, “called the null model”, was fitted without explanatory variables to test random variability in the intercept and to estimate the intra‐class correlation coefficient (ICC), and Proportion Change in Variance (PCV), (2) Model I was fitted to examine the effects of individual‐level variables on the outcome interest, (3) Model II was also fitted to examine the effect of community‐level predictors on the outcome of interest and (4) the final Model III, “called the full model”, was fitted to examine the effects of both individual and community‐level independent variables on the outcome of interest simultaneously.

**FIGURE 1:**
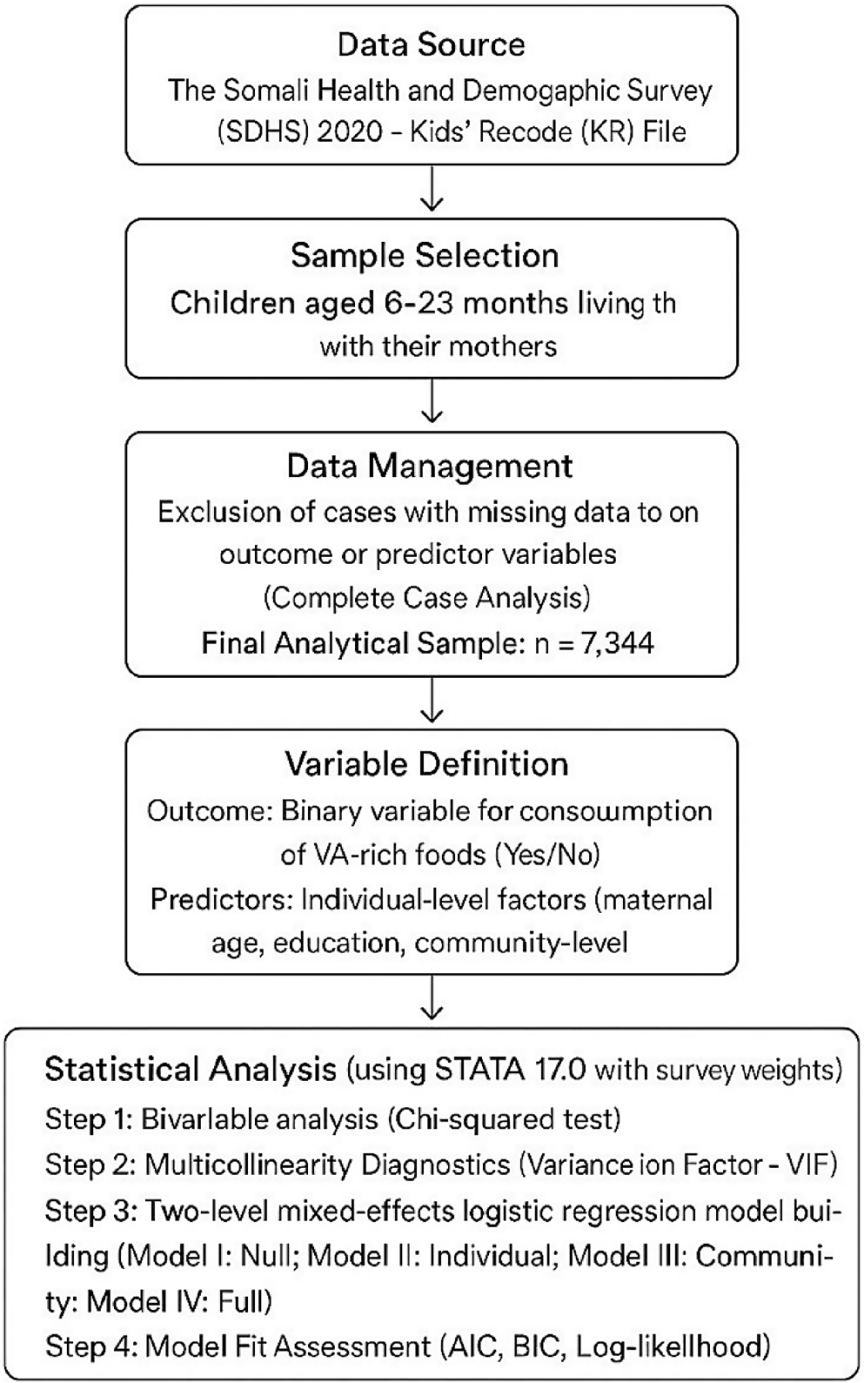
Flowchart of this study.

Both Akaike's Information Criterion (AIC) and Bayesian Information Criterion (BIC) were used to compare the four models, alongside log‐likelihood values. Lower AIC and BIC values indicate a better trade‐off between model fit and complexity. While AIC is often favored for its properties in selecting models optimal for prediction and tends to be less stringent in penalizing complexity with large sample sizes (such as *N* = 7344 in this study), BIC favors more parsimonious models by imposing a stronger penalty for additional parameters. The flowchart of this study is presented in Figure 1.

## 3 Results

### 3.1 Socio‐Demographic Characteristics of the Study Population, Somalia (SHDS), 2020

Table 1 presents the socio‐demographic characteristics of the study population in Somalia. Approximately 38.90% of children consumed VA‐rich foods, while 61.10% did not. Participants were primarily aged 20–29 years (48.28%), with the age group 25–29 being the largest (27.64%). The age group 15–19 comprised 6.40% of the population. The population was distributed across several regions, with the highest representation from Banadir (12.45%), followed by Togdheer (7.67%) and Sanaag (8.27%). Awdal had the lowest representation at 4.71%. The majority of respondents resided in rural areas (40.44%), with urban residents accounting for 25.84% and nomadic residents for 33.71%. A significant portion of the population had no formal education (83.91%), while only 12.51% had completed primary education and 3.58% had secondary education.

**TABLE 1:** Socio‐demographic characteristics of the study population, Somalia (SHDS), 2020.

Variables	Labels	Status of Vitamin A consumption		*p* of *χ* ^2^
		Consumed VA‐rich foods *n* (%)	Did not consume VA‐rich foods *n* (%)	
Age (years)	15–19	126 (26.81)	344 (73.19)	< 0.001
	20–24	524 (34.56)	992 (65.44)	
	25–29	785 (38.67)	1245 (61.33)	
	30–34	604 (41.14)	864 (58.86)	
	35–39	517 (42.10)	711 (57.90)	
	40–44	221 (46.72)	252 (53.28)	
	45–49	80 (50.31)	79 (49.69)	
Region	Awdal	178 (51.45)	168 (48.55)	< 0.001
	Woqooyi Galbeed	285 (55.02)	233 (44.98)	
	Togdheer	210 (37.30)	353 (62.70)	
	Sool	94 (15.80)	501 (84.20)	
	Sanaag	153 (25.21)	454 (74.79)	
	Bari	104 (33.23)	209 (66.77)	
	Nugaal	113 (30.54)	257 (69.46)	
	Mudug	176 (43.67)	227 (56.33)	
	Galgaduud	116 (25.00)	348 (75.00)	
	Hiraan	173 (38.53)	276 (61.47)	
	Middle Shabelle	156 (39.80)	236 (60.20)	
	Banadir	669 (73.19)	245 (26.81)	
	Bay	75 (43.60)	97 (56.40)	
	Bakool	115 (27.85)	298 (72.15)	
	Gedo	134 (33.09)	271 (66.91)	
	Lower Juba	106 (25.24)	314 (74.76)	
Education	No Education	2235 (36.27)	3927 (63.73)	< 0.001
	Primary	462 (50.27)	457 (49.73)	
	Secondary	160 (60.84)	103 (39.16)	
Wealth status	Lower income	743 (23.28)	2448 (76.72)	< 0.001
	Middle	1347 (46.23)	1567 (53.77)	
	Higher income	767 (61.90)	472 (38.10)	
Marital status	Married	2547 (38.20)	4120 (61.80)	< 0.001
	Divorced	310 (45.79)	367 (54.21)	
Media exposure	Yes	83 (68.60)	38 (31.40)	< 0.001
	No	2774 (38.41)	4449 (61.59)	
Preceding birth interval (in months)	< 24	694 (41.46)	980 (58.54)	0.015
	≥ 24	2163 (38.15)	3507 (61.85)	
Current age in months	6–11	50 (10.46)	428 (89.54)	< 0.001
	12–17	106 (34.75)	199 (65.25)	
	18–23	2701 (41.17)	3860 (58.83)	
Parity	1	242 (28.44)	609 (71.56)	< 0.001
	2–4	1897 (39.57)	2897 (60.43)	
	5+	718 (42.26)	981 (57.74)	
Giving Vitamin A supplement in the last 6 months	Yes	703 (72.25)	270 (27.75)	< 0.001
	No	2151 (33.82)	4209 (66.18)	
Place of delivery	Home	1919 (34.51)	3641 (65.49)	< 0.001
	Health facility	938 (52.58)	846 (47.42)	
ANC visits attended during pregnancy	No ANC visit	1490 (31.84)	3189 (68.16)	< 0.001
	1	200 (43.20)	263 (56.80)	
	2–3	817 (51.94)	756 (48.06)	
	4+	350 (55.64)	279 (44.36)	
breastfeeding status	Yes	932 (25.55)	2716 (74.45)	< 0.001
	No	1925 (52.08)	1771 (47.92)	
Source of drinking water	Unimproved	1684 (41.33)	2391 (58.67)	< 0.001
	Improved	1173 (35.88)	2096 (64.12)	
Drugs for intestinal parasites during pregnancy	Yes	245 (53.49)	213 (46.51)	< 0.001
	No	2612 (37.93)	4274 (62.07)	
Getting medical help for self: distance to the health facility	Yes	1648 (35.76)	2961 (64.24)	< 0.001
	No	1208 (44.22)	1524 (55.78)	

Most participants were classified as lower income (43.45%), followed by those in the middle wealth status (39.68%) and higher income (16.87%). A substantial number of women (63.71%) reported no antenatal care visits during pregnancy, with only a small percentage attending four or more visits (8.56%). The majority (62.78%) reported having access to medical help, indicating a favorable perception of distance to health facilities. Media exposure was minimal, with only 1.65% having access, while a significant majority (76.32%) could not read. More than half of the respondents relied on unimproved drinking water sources (55.49%). Breastfeeding rates were nearly equal, with 49.67% breastfeeding and 50.33% not. The majority of participants were married (90.78%). Most women (77.21%) had a preceding birth interval of 24 months or more. The current age of children primarily fell within the 18–23 months category (89.34%). A significant proportion of deliveries occurred at home (75.71%). Additionally, the use of drugs for intestinal parasites during pregnancy was low (6.24%), and a majority (86.73%) had not received vitamin A supplements in the past 6 months. Most participants had 2–4 children (65.28%), with 11.59% having one child and 23.13% having five or more (Table 1).

**FIGURE 2:**
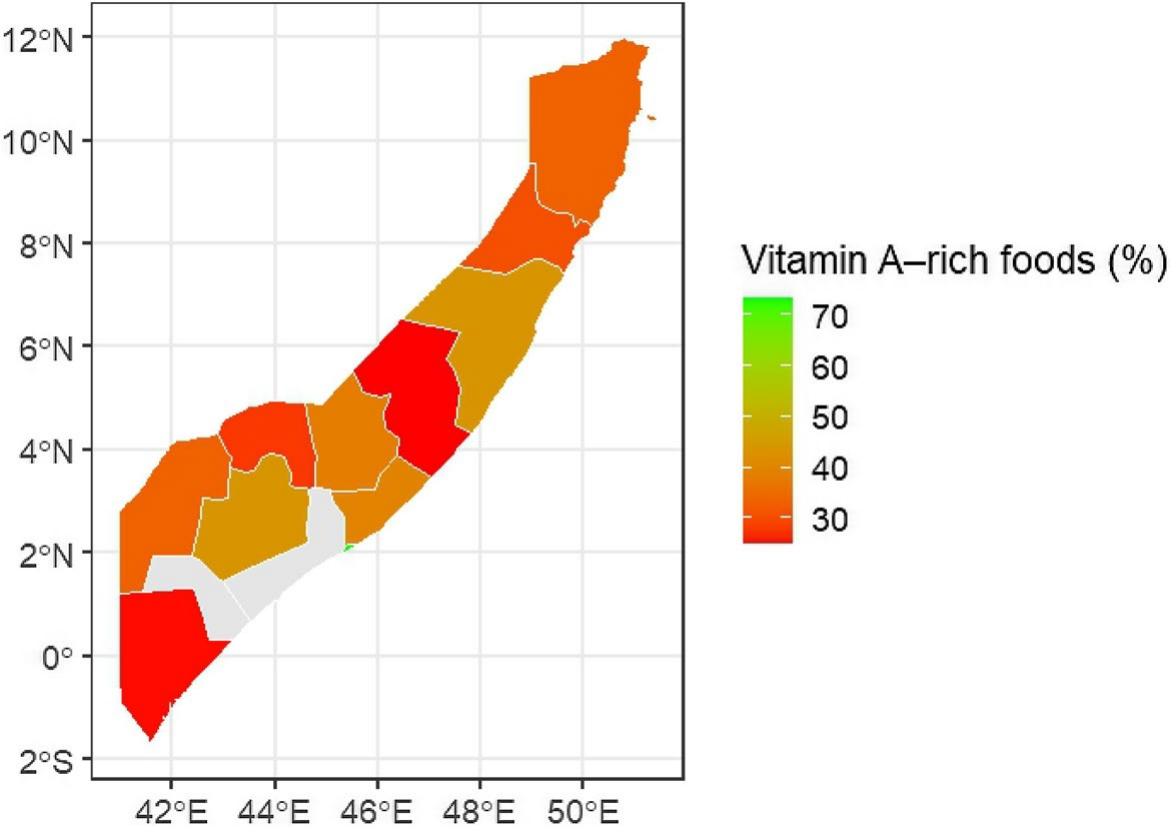
Spatial distribution of consumed VA‐rich foods.

Figure 2 illustrates the Spatial distribution of consumed VA‐rich foods. Findings depict that Banadir (73.2%) has the highest prevalence of consuming VA‐rich foods, followed by Woqooyi Galbeed (55.0%) and Awdal (51.5%). The lowest prevalence of consuming VA‐rich foods is observed in Sool (15.8%), Sanaag (25.2%), and Lower Juba (25.2%). The northern urban regions (Awdal, Woqooyi Galbeed) and the capital region (Banadir) show notably higher consumption of vitamin A–rich foods. Moreover, central and southern pastoral/agrarian regions (Galgaduud, Hiraan, Bakool, Gedo, Lower Juba) have lower intake levels, likely reflecting limited dietary diversity and food insecurity.

### 3.2 Multicollinearity Diagnostics

To evaluate the robustness of our final multilevel logistic regression model (Model III), multicollinearity was assessed using Variance Inflation Factors (VIF) for each independent variable. Multicollinearity can inflate the variance of regression coefficients, potentially leading to unreliable or unstable estimates. A common rule of thumb suggests that VIF values greater than 10, or sometimes a more conservative threshold of 5, indicate problematic multicollinearity. The results of this diagnostic check are presented in Table S1. All individual VIF values were substantially below the commonly cited threshold of 10, and also well below the more conservative threshold of 5. These results strongly indicate that multicollinearity was not a significant issue among the predictor variables.

### 3.3 Bivariate and Multivariate Analysis of Determinants of Consuming VA‐Rich Foods Among Mothers of Children Aged 6–23 Months in Somalia (*n* = 7344)

Table 2 presents the results of multivariable analysis, including crude odds ratios (COR) and adjusted odds ratios (AOR) with 95% confidence intervals (CI) for different demographic and socioeconomic variables. The results indicate that mothers aged 20–24 years have a lower likelihood of consuming VA‐rich foods (COR = 0.69, AOR = 1.13), while those aged 40–44 years show a significant decrease in odds (COR = 0.40, AOR = 0.66). Notably, mothers aged 45–49 years exhibit the lowest odds of consuming VA‐rich foods (COR = 0.36, AOR = 0.63). Mothers with primary (AOR = 0.56) and secondary education (AOR = 0.52) show significantly lower odds of consuming VA‐rich foods compared to those with no education. Compared to rural mothers, nomadic mothers have a higher likelihood of consuming VA‐rich foods (COR = 0.60, AOR = 0.61), while urban mothers show no significant difference (COR = 1.01, AOR = 0.97). There are substantial regional variations. For instance, mothers in the Sool region have a very high COR (5.60) for good consumption, while those in Banadir show reduced odds (COR = 0.38, AOR = 1.80).

**TABLE 2:** Multivariable analysis of determinants of consuming VA‐rich foods among mothers of children aged 6–23 months in Somalia (*n* = 7344).

Variable	COR 95% CI	AOR 95% CI
**Age (years)**		
15–19	Ref	Ref
20–24	0.69 (0.55–0.87)*	1.13 (0.85–1.5)
25–29	0.58 (0.46–0.72)*	0.9 (0.70–1.24)
30–34	0.5 (0.41–0.65)*	0.8 (0.61–1.11)
35–39	0.5 (0.39–0.6)*	0.80 (0.58–1.09)
40–44	0.4 (0.31–0.54)*	0.66 (0.46–0.95)*
45–49	0.36 (0.24–0.5)*	0.63 (0.40–0.99)*
**Residence**		
Rural	Ref	Ref
Urban	1.01 (0.89–1.14)	0.97 (0.83–1.13)
Nomadic	0.6 (0.53–0.68)*	0.61 (0.5–0.71)*
**Region**		
Awdal	Ref	Ref
Woqooyi Galbeed	0.86 (0.659–1.13)	1.1 (0.82–1.48)
Togdheer	1.78 (1.35–2.33)*	0.91 (0.68–1.22)
Sool	5.6 (4.16–7.6)*	1.2 (0.90–1.6)
Sanaag	3.14 (2.37–4.15)*	1.36 (1.02–1.8)*
Bari	2.1 (1.5–2.9)*	3.38 (2.44–4.68)*
Nugaal	2.4 (77–3.27)*	3.4 (2.50–4.89)*
Mudug	1.36 (1.02–1.82)*	3.2 (2.3–4.5)*
Galgaduud	3.17 (2.36–4.28)*	2.39 (1.7–3.34)*
Hiraan	1.6 (1.27–2.24)*	2.87 (2.04–4.0)*
Middle Shabelle	1.6 (1.19–2.14)*	2.2 (1.62–3.13)*
Banadir	0.38 (0.30–0.5)*	1.8 (1.3–2.4)*
Bay	1.37 (0.94–1.97)*	3.3 (2.06–5.3)*
Bakool	2.7 (2.03–3.7)*	2.29 (1.67–3.14)*
Gedo	2.1 (1.5–2.8)*	1.7 (1.2–2.4)*
Lower Juba	3.1 (2.3–4.25)*	1.89 (1.38–20.6)*
**Education**		
No education	Ref	
Primary	0.56 (0.48–0.64)*	0.56 (0.47–0.66)*
Secondary	0.36 (0.28–0.47)*	0.52 (0.38–0.72)*
**Wealth status**		
Lower income	Ref	
Middle	0.35 (0.31–0.39)*	0.43 (0.37–0.49)*
Higher income	0.19 (0.16–0.21)*	0.28 (0.23–0.33)*
**Marital status**		
Married	Ref	Ref
Divorced	0.73 (0.62–0.85)*	0.82 (0.66–1.03)
**Media exposure**		
Yes	Ref	Ref
No	3.5 (2.3–5.15)*	1.98 (1.28–3.06)*
**Preceding birth interval (in months)**		
< 24	Ref	Ref
≥ 24	1.14 (1.02–1.28)*	0.99 (0.87–1.1)
**Child's current age (months)**		
6–11	Ref	
12–17	0.21 (0.15–0.32)*	0.26 (0.17–0.39)*
18–23	0.16 (0.12–0.22)*	0.21 (0.14–0.30)*
**Parity**		
1	Ref	Ref
2–4	0.60 (0.51–0.71)*	0.93 (0.73–1.17)
5+	0.54 (0.45–0.64)*	1.0 (0.78–1.37)
**Giving Vitamin A supplements in the last 6 months**		
Yes	Ref	
No	5.0 (4.3–5.9)*	3.49 (2.9–4.1)*
**Place of delivery**		
Home	Ref	Ref
Health facility	0.47 (0.42–0.52)*	0.76 (0.6–0.88)*
**ANC visits attended during pregnancy**		
0	Ref	Ref
1	0.6 (0.50–0.74)*	0.69 (0.56–0.87)*
2–3	0.43 (0.38–0.48)*	0.61 (0.53–0.71)*
4+	0.37 (0.31–0.44)*	0.64 (0.51–0.79)*
**Breastfeeding status**		
Yes	Ref	Ref
No	0.3 (0.28–0.35)*	0.36 (0.32–0.40)*
**Source of drinking water**		
Unimproved	Ref	Ref
Improved	1.2 (1.1–1.38)*	0.88 (0.76–1.0)
**Drugs for intestinal parasites during pregnancy**		
Yes	Ref	Ref
No	1.8 (1.5–2.27)*	1.1 (0.93–1.48)
**Getting medical help for oneself: distance to the health facility**		
Yes	Ref	Ref
No	0.7 (0.63–0.77)*	0.77 (0.69–0.86)*

*Note:* Ref: Reference Category; *p < 0.05.

Abbreviations: AOR, Adjusted odds ratios (AOR); COR, Crude odds ratios.

Mothers in the rich category have significantly higher odds (COR = 0.186, AOR = 0.28) compared to those who have a lower income. Divorced mothers show a decreased likelihood of consuming VA‐rich foods (AOR = 0.82) compared to married mothers. Mothers with media exposure are significantly more likely to consume VA‐rich foods (AOR = 1.98) compared to those without. Mothers with a preceding birth interval of 24 months or more do not show significant differences in consumption (AOR = 0.99). The likelihood of consuming VA‐rich foods increases with the age of the child, with the highest odds for children aged 18–23 months (AOR = 0.21). Mothers with 2–4 children have a slight increase in odds (AOR = 0.93), but those with 5 or more children show no significant difference. Mothers who provided vitamin A supplements in the last 6 months have significantly higher odds of good consumption (AOR = 3.49). Delivery at a health facility compared to home significantly increases the odds of consuming VA‐rich foods (AOR = 0.76). Increased attendance at antenatal care visits correlates with higher odds of good vitamin A consumption, particularly for those attending 4 or more visits (AOR = 0.64). Non‐breastfeeding mothers have significantly lower odds of consuming VA‐rich foods (AOR = 0.36). Mothers using unimproved water sources show slightly lower odds (AOR = 0.88), while those who report distance to health facilities as a barrier exhibit reduced odds (AOR = 0.77).

### 3.4 Determinants of Consuming VA‐Rich Foods Among Mothers of Children Aged 6–23 Months in Somalia

**FIGURE 3:**
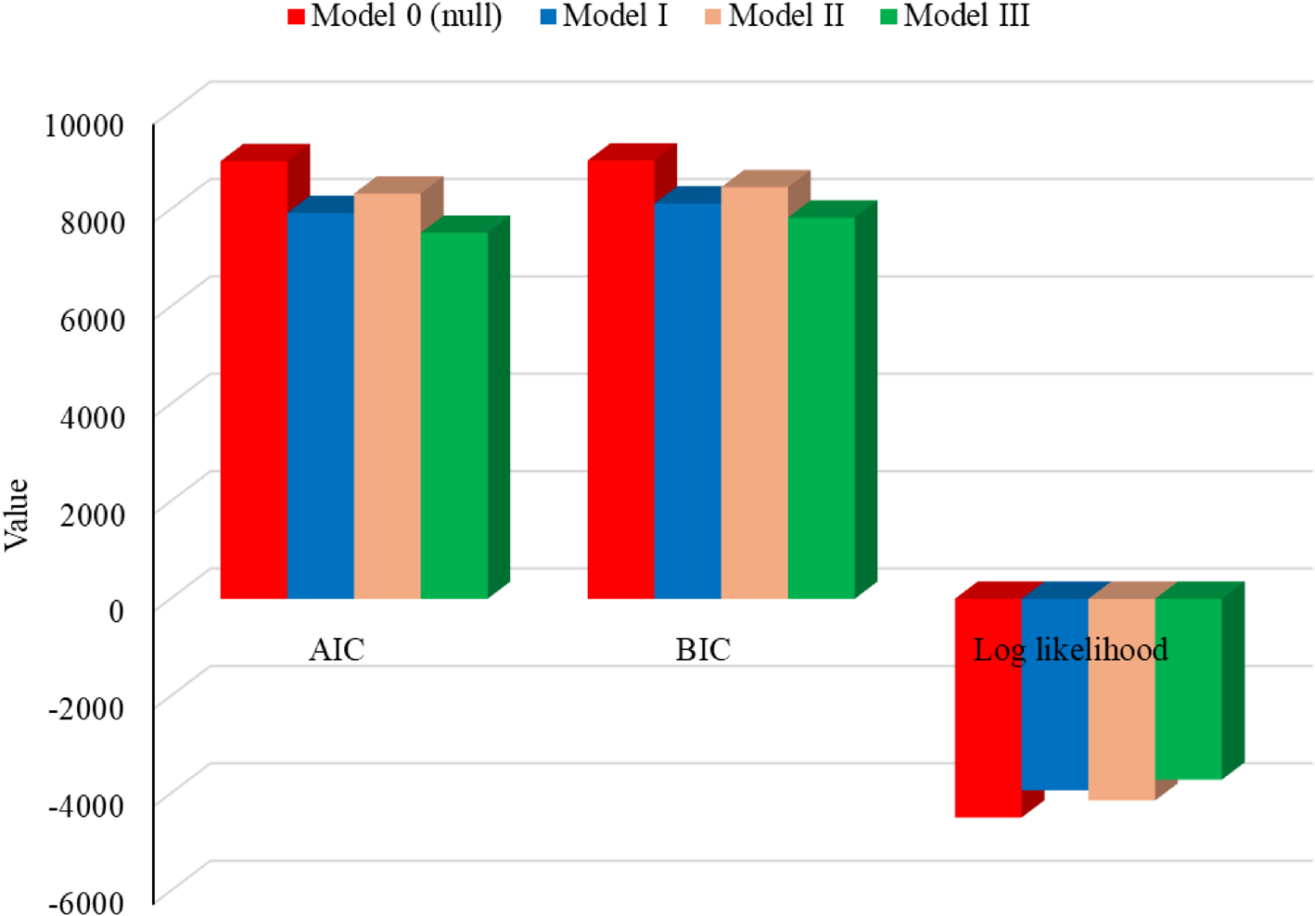
Results of model evaluation criteria.

In our analysis, both AIC and BIC consistently indicated that Model III, the full model, was the best‐fitted model for this data (Table 3, Figure 3). It achieved the lowest AIC (7518.327) and the lowest BIC (7828.815) among the models assessed, indicating the most optimal balance between model fit and parsimony according to both criteria. The log‐likelihood values also support this conclusion, with Model III having the least negative (closest to zero) log‐likelihood (−3714.16).

**TABLE 3:** Multilevel multivariable logistic regression models on individual and community‐level determinants associated with consuming VA‐rich foods in mothers of children aged 6–23 months in Somalia.

Variables	Model 0 empty model	Model I (individual‐level variables) AOR (95% CI)	Model II (community‐level variables) AOR (95% CI)	Model III (both individual and community‐level variables) AOR (95% CI)
**Age**				
15–19		1 (ref)		1 (ref)
20–24		1.1 (0.84–1.52)		1.08 (0.79–1.46)
25–29		0.95 (0.708–1.29)		0.91 (0.67–1.24)
30–34		0.85 (0.62–1.17)		0.83 (0.60–1.14)
35–39		0.79 (0.57–1.09)		0.75 (0.54–1.05)
40–44		0.59 (0.41–0.86)*		0.59 (0.40–0.87)*
45–49		0.55 (0.34–0.89)*		0.54 (0.33–0.88)
**Residence**				
Rural			1 (ref)	1 (ref)
Urban			1.2 (1.0–1.5)*	1.1 (0.94–1.47)
Nomadic			6.7 (5.4–8.3)*	4.0 (3.0–5.25)*
**Region**				
Awdal			I (ref)	1 (ref)
Woqooyi Galbeed			1.0 (0.75–1.48)	1.0 (0.73–1.5)
Togdheer			2.1 (1.5–2.99)*	2.0 (1.4–2.9)*
Sool			6.8 (4.7–9.8)*	6.5 (4.40–9.69)*
Sanaag			4.2 (2.99–6.0)*	3.7 (2.56–5.5)*
Bari			2.9 (2.0–4.4)*	2.6 (1.74–4.07)*
Nugaal			3.1 (2.18–4.66)*	2.8 (1.8–4.3)*
Mudug			1.6 (1.1–2.35) *	1.49 (1.0–2.2)*
Galgaduud			4.70 (3.2–6.85)*	4.3 (2.8–6.49)*
Hiraan			2.6 (1.82–3.74)*	1.8 (1.2–2.68)*
Middle Shabelle			2.36 (1.6–3.38)*	1.6 (1.12–2.48)*
Banadir			0.98 (0.70–1.37)	0.96 (0.66–1.40)
Bay			3.8 (2.27–6.6)*	3.2 (1.8–5.75)*
Bakool			3.9 (2.7–5.77)*	2.96 (1.9–4.49)*
Gedo			2.9 (2.0–4.2)*	2.27 (1.5–3.38)*
Lower Juba			5.7 (3.9–8.49)*	4.42 (2.9–6.74)*
**Education**				
No education		1 (ref)		1 (ref)
Primary		0.8 (0.69–0.98)*		0.76 (0.64–0.91)*
Secondary		0.80 (0.59–1.1)		0.79 (0.57–1.09)
**Wealth status**				
Lower income		1 (ref)		1 (ref)
Middle		0.51 (0.434–0.598)*		0.82 (0.68–0.98)*
Higher income		0.29 (0.23–0.365)		0.56 (0.45–0.711)*
**Marital status**				
Married		1 (ref)		1 (ref)
Divorced		0.97 (0.80–1.17)		0.98 (0.80–1.19)
**Media exposure**				
Yes		1 (ref)		1 (ref)
No		1.5 (0.99–2.46)*		1.3 (0.87–2.15)
**Preceding birth interval (in months)**				
< 24		1 (ref)		1 (ref)
≥ 24		0.9 (0.86–1.13)		0.97 (0.84–1.12)
**Current age in months**				
6–11		1 (ref)		1 (ref)
12–17		0.26 (0.17–0.40)*		0.24 (0.15–0.37)*
18–23		0.19 (0.13–0.276)*		0.17 (0.12–0.25)*
**Parity**				
1		1 (ref)		1 (ref)
2–4		0.97 (0.76–1.25)		1.0 (0.78–1.29)
5+		1.17 (0.88–1.58)		1.25 (0.92–1.68)
**Giving Vitamin A supplement in last 6 months**				
Yes		1 (ref)		1 (ref)
No		3.1 (2.6–3.7)*		2.8 (2.42–3.4)*
**Place of delivery**				
Home		1 (ref)		1 (ref)
Health facility		0.85 (0.73–0.996)*		0.9 (0.85–1.15)
ANC visits attended during pregnancy				
No ANC visit		1 (ref)		**1 (ref)**
1		0.72 (0.57–0.910)*		0.78 (0.62–0.99)*
2–3		0.68 (0.59–0.798)*		0.8 (0.69–0.94)*
4+		0.7 (0.58–0.90)		0.92 (0.74–1.16)*
**Breastfeeding status**				
Yes		1 (ref)		1 (ref)
No		0.35 (0.31–0.396)*		0.37 (0.32–0.41)*
**Source of drinking water**				
Unimproved		1 (ref)		1 (ref)
Improved		1.1 (1.0–1.29)*		0.98 (0.84–1.13)
**Drugs for intestinal parasites during pregnancy**				
Yes		1 (ref)		1 (ref)
No		1.1 (0.88–1.4)		1.0 (0.86–1.39)
**Getting medical help for self: distance to the health facility**				
Yes		1 (ref)		1 (ref)
No		0.91 (0.81–1.03)		0.89 (0.79–1.0)
Random effect				
Community‐level Variance (SE)	1.9 (0.227)	0.62 (0.1136)	0.26 (0.063)	0.22 (0.064)
ICC (95%)	3.6%	1.5%	0.74%	0.17%

Abbreviation: ref., Reference category.

Table 3 summarizes the adjusted odds ratios (AORs) and 95% confidence intervals (CIs) derived from four logistic regression models. The results indicate a significant community‐level variance of 1.9 (SE = 0.227), with an intra‐class correlation coefficient (ICC) of 3.6%. This suggests that a small but notable proportion of the variation in the consumption of VA‐rich foods is attributable to differences at the community‐level. Model I integrates individual‐level variables into the analysis. Findings reveal that mothers aged 40–44 (AOR = 0.59, 95% CI: 0.41, 0.86) and 45–49 (AOR = 0.55, 95% CI: 0.34, 0.89) have significantly lower odds of adequately consuming VA‐rich foods compared to the reference group, mothers aged 15–19 years. Nomadic mothers are observed to have substantially higher odds of consuming VA‐rich foods (AOR = 6.7, 95% CI: 5.4, 8.3) compared to rural mothers. Additionally, mothers with primary education demonstrate reduced odds of consuming adequate VA‐rich foods (AOR = 0.80, 95% CI: 0.69, 0.98) when compared to mothers with no education (the reference category). Socioeconomic status also emerges as a significant determinant, with mothers in middle‐income (AOR = 0.5, 95% CI: 0.43, 0.60) and higher income households (AOR = 0.29, 95% CI: 0.23, 0.37) exhibiting lower odds of consuming VA‐rich foods compared to mothers in lower income households.

Model II incorporates community‐level variables, alongside the individual‐level predictors. The results generally align with those of Model I, with minor variations in the odds ratios. Notably, the region of Sool is identified as a significant determinant of consuming VA‐rich foods (AOR = 6.8, 95% CI: 4.7, 9.8). Model III combines both individual and community‐level variables, resulting in further adjustments to the odds ratios. For example, mothers who have consumed VA‐rich foods for their children within the last 6 months are strongly associated with higher odds of adequate vitamin A consumption (AOR = 3.1, 95% CI: 2.6, 3.7). Across the four models, community‐level variance decreases progressively. In Model III, the community variance declines to 0.22 (SE = 0.064), with an ICC of 0.17%. This reduction suggests that individual‐level variables account for a substantial portion of the variance in consuming VA‐rich foods, underscoring the importance of individual‐level determinants in explaining the observed patterns of vitamin A intake among mothers in Somalia.

## 4 Discussion

The findings reveal a complex interplay of individual‐ and community‐level factors influencing VA intake, highlighting the need for multifaceted interventions to address the persistent challenge of vitamin A deficiency (VAD) in Somalia. The research findings indicate that a substantial proportion of children in Somalia (61.1%) did not consume VA‐rich foods. This is consistent with previous research highlighting the alarmingly high prevalence of VAD in Somalia (FSNAU n.d.) and underscores the significant public health implications of inadequate VA intake, contributing to increased morbidity and mortality among young children (Amimo et al. 2022; Penkert et al. 2021; Gordon et al. 2013; Stephenson et al. 2000; du Plessis et al. 2007). The high prevalence of VAD in the Somali context is further exacerbated by factors such as limited access to VA‐rich foods due to income inequality and limited agricultural production (Dharod et al. 2011; Fekadu et al. 2015; Kalid et al. 2019). Older mothers (40–49 years) showed significantly lower odds of consuming VA‐rich foods compared to younger mothers (15–19 years). This could be attributed to factors such as reduced knowledge about nutrition or decreased capacity for engaging in food preparation and provision. Nomadic mothers, in contrast, demonstrated significantly higher odds of consuming VA‐rich foods than their rural counterparts. This may reflect differences in dietary practices or access to specific food sources within nomadic communities. Mothers with primary education exhibited lower odds of consuming VA‐rich foods than those with no formal education, potentially suggesting a complex relationship between education and nutritional knowledge, or socioeconomic factors influencing access to nutritional resources. Wealthier mothers showed a higher likelihood of consuming VA‐rich foods, reinforcing the link between socioeconomic status and access to nutritious foods. Importantly, mothers who provided vitamin A supplements in the last 6 months had significantly higher odds of consuming VA‐rich foods, confirming the effectiveness of supplementation programs (du Plessis et al. 2007; Low et al. 2007). However, challenges related to low coverage of these programs remain (Ali et al. 2022), consistent with our data revealing low supplementation coverage. Furthermore, breastfeeding status was strongly associated with better consumption of VA‐rich foods, highlighting the importance of promoting and supporting breastfeeding practices (World Health Organization (WHO) 2011).

Community‐level factors also played a significant role. The Sool region stood out as having significantly higher odds of consuming VA‐rich foods, suggesting regional variations in food security, cultural practices, or access to health services that warrant further investigation (Oumer et al. 2020; Demsash et al. 2022; Raut et al. 2019; Amimo et al. 2022; Gilano et al. 2021). By accounting for both individual and community‐level variables, our model provides a more comprehensive and context‐specific understanding of the factors influencing the consumption of VA‐rich foods in Somalia. Our results also contribute to the ongoing debate on the effectiveness of interventions to combat VAD (Oresanya et al. 2022), highlighting the need for comprehensive interventions that extend beyond supplementation programs to address individual and contextual factors that impact diet and nutritional outcomes. These findings have strong implications for achieving several Sustainable Development Goals (SDGs). Specifically, the study directly relates to SDG 2 (Zero Hunger) and SDG 3 (Good Health and Well‐being) by highlighting the significant challenge of VAD in Somalia and identifying key determinants of adequate consumption of VA‐rich foods. Addressing the factors identified in this study—improving access to VA‐rich foods, enhancing nutritional knowledge through education, strengthening health systems, and ensuring equitable access to supplementation programs—is crucial for reducing childhood malnutrition and improving child health outcomes (Demsash et al. 2022). The socioeconomic disparities observed emphasize the need for targeted interventions that consider the vulnerability of different groups within the Somali population. This highlights the interconnectedness of SDGs, as addressing issues such as poverty (SDG 1) and inequalities in access to healthcare (SDG 10) is essential for achieving progress towards better nutrition and child health.

### 4.1 Limitations

The cross‐sectional nature of the data limits the establishment of causal relationships. Further research employing longitudinal studies and qualitative methods is needed to explore the identified associations in more detail. The reliance on self‐reported dietary data can also introduce recall bias. To verify, future studies should consider employing objective measures of VA intake when feasible. Future work will involve spatial analysis to generate more insightful findings.

## 5 Conclusion

The results indicate a multifaceted interaction between individual and community‐level determinants that profoundly affect consuming VA‐rich foods, thereby emphasizing the necessity for comprehensive and contextually relevant interventions to tackle the ongoing issue of vitamin A deficiency (VAD). Moreover, breastfeeding status demonstrated a significant positive correlation, highlighting the essential necessity to promote and sustain optimal breastfeeding practices. Addressing socioeconomic inequalities and advancing nutritional literacy through educational initiatives is vital for fostering sustainable dietary enhancements. The observable regional disparities emphasize the critical requirement for individualized strategies that are adapted to local conditions.

## Author Contributions

**Abdirahman Omer Ali:** conceptualization (equal), formal analysis (equal), writing – original draft (equal). **Abdisalam Mahdi Hassan:** conceptualization (equal), data curation (equal), formal analysis (equal), writing – original draft (equal). **Hawa Dahir Mahamoud:** data curation (equal), writing – original draft (equal). **Nimco Mahamoud Barakale:** methodology (equal), writing – original draft (equal). **Muhyadin Yusuf Dahir:** formal analysis (equal), methodology (equal), writing – original draft (equal). **Md. Moyazzem Hossain:** methodology (equal), supervision (equal), validation (equal), visualization (equal), writing – review and editing (equal).

## Funding

The authors have nothing to report.

## Ethics Statement

This study is based on a secondary dataset, and the initial survey was conducted with proper ethical approval from the respective authorities.

## Consent

The authors have nothing to report.

## Conflicts of Interest

The authors declare no conflicts of interest.

## Supporting information


**Table S1:** Variance Inflation Factor (VIF) and Tolerance (1/VIF) for predictor variables in the final multilevel model (Model IV).

## Data Availability

The data is freely accessible through the Somali National Data Archive (SoNADA) website at https://microdata.nbs.gov.so/index.php/catalog/50.
