# Supplementary material for: Determinants of Vitamin A Consumption Among Children Aged 6–23 Months in Somalia: A Multilevel Analysis of SDHS 2020
Source: Food Sci Nutr. 2025 Dec 8;13(12):e71301. doi: 10.1002/fsn3.71301 (PMC12683356; doi:10.1002/fsn3.71301)
Supplement: Supplementary file 1 — Table S1: Variance Inflation Factor (VIF) and Tolerance (1/VIF) for predictor variables in the final multilevel model (Model IV). [file FSN3-13-e71301-s001.docx]

**Supplementary Table 1:** Variance Inflation Factor (VIF) and Tolerance (1/VIF) for Predictor Variables in the Final Multilevel Model (Model IV)

| **Variable Code** | **VIF** | **Tolerance (1/VIF)** |
| --- | --- | --- |
| V190 | 1.73 | 0.577203 |
| V013 | 1.64 | 0.608537 |
| V159 | 1.52 | 0.659546 |
| V201 | 1.51 | 0.663014 |
| V102 | 1.50 | 0.666502 |
| V171 | 1.40 | 0.713739 |
| V158 | 1.36 | 0.734208 |
| V157 | 1.32 | 0.756573 |
| V024 | 1.32 | 0.759528 |
| M1G | 1.28 | 0.782616 |
| M14 | 1.25 | 0.798715 |
| M15 | 1.24 | 0.804209 |
| B19 | 1.24 | 0.804401 |
| V025 | 1.24 | 0.807303 |
| V106 | 1.21 | 0.828883 |
| V113 | 1.16 | 0.864043 |
| V404 | 1.14 | 0.875420 |
| H34 | 1.11 | 0.901710 |
| B11 | 1.10 | 0.905098 |
| V169B | 1.05 | 0.950406 |
| V501 | 1.04 | 0.962374 |
| V151 | 1.02 | 0.983259 |
| **Mean VIF** | **1.29** |  |
|  |  |  |

Note: Variable codes correspond to the DHS variable names for predictors. These include: V190 (Wealth status), V013 (Maternal age group), V159 (Media exposure – specific, e.g., listens to radio), V201 (Parity/Total children ever born), V102 (Type of place of residence – urban/rural, often used for rural if V025 covers urban/rural/nomadic), V171 (Maternal education level – might be a more detailed version if V106 is primary/secondary/none), V158 (Media exposure – specific, e.g., watches TV), V157 (Media exposure – specific, e.g., reads newspaper), V024 (Region), M1G (Child's current age in months), M14 (Number of ANC visits), M15 (Place of delivery), B19 (Preceding birth interval), V025 (Type of place of residence – urban/rural/nomadic), V106 (Maternal education), V113 (Source of drinking water), V404 (Perceived distance to health facility), H34 (Child received Vitamin A supplement in last 6 months), B11 (Breastfeeding status), V169B (Drugs for intestinal parasites during pregnancy), V501 (Marital status), V151 (Respondent's employment status – please verify if this variable was indeed in your final Model IV; if not, remove it from the VIF table or clarify). Authors should ensure these mappings accurately reflect the variables used in their final Model IV presented in Table 3.
